# Toxicological risk at workplace and toxicity as Life Cycle Assessment impact category: Substitution of solvents as an example

**DOI:** 10.17179/excli2016-764

**Published:** 2017-01-10

**Authors:** Thomas Schupp, Philipp Alexander Georg, Guenter Kirstein

**Affiliations:** 1 Muenster University of Applied Science, Chemical Engineering, Stegerwaldstrasse 39, D-48565 Steinfurt, Germany; 2 ALGURA Chemie GmbH Co KG, Handwerkerstraße 12, D-48720 Rosendahl, Germany

**Keywords:** life cycle assessment, toxicological risk, worker protection, substitution

## Abstract

Substitution of hazardous substances against less hazardous ones is a central requirement of the European Chemical Regulation REACH (European Regulation 1907/2006/EC). Hazardous substances emitted from products may not only affect the worker; drift off and distribution in the environment may finally result in exposure of the general population. This potential threat to health is covered by the impact category “toxicity” in Life Cycle Assessments. In this paper, we present a case of a substitution of volatile organic compounds in a reactive varnish, and compare the “old” formulation with the “new” formulation against health risk to the worker, and concerning the Life Cycle Assessment impact category “toxicity”. The “old” formulation contained Naphtha (petroleum), hydrodesulfurized, heavy and Solvent naphtha (petroleum), light, aromatic. In the new formulation, both naphthas were replaced by n-Butylacetate, 1-Ethoxy-2-propyl acetate and Ethyl-3-ethoxy propionate. In the European Union, the naphthas are classified as mutagens and carcinogens category 1, officially. However, if benzene is below 0.1 %, registrants in the EU proposed to omit this classification, and todays naptha products on the market obviously have benzene contents below 0.1 %. On a first glance, the improvement for workplace safety introduced by the substitution, therefore, is comparatively small, as it is for toxicity in Life Cycle Assessment. However, when background knowledge concerning chemical production processes of naphtha is included, benzene below a content of 0.1 % needs to be taken into consideration, and the benefit of substitution is more obvious.

## Introduction

Varnishes typically contain solvents that not only ensure easy spreading and brushing on surfaces; a fine orchestrated evaporation process ensures hardening of the varnish with release of an optical and mechanical optimized surface. Due to the purpose the solvent serves, exposure to volatile organic compounds is unavoidable. For the sake of worker protection, employers are requested to look for substitutions for solvents with a relatively high risk potential against others with a lower risk potential. 

Life Cycle Assessment according to standards ISO 14040 and ISO 14044 assesses the total environmental impact of a product or service. In that respect, the benchmark for comparison of products is the “functional unit” (fu), i. e. the service of the product. For example, the functional unit for a varnish could be “protection of 1 m² wood against weathering for 10 years”. To fulfil this requirement, varnish A has to be applied in an amount of X kg per m², whereas from varnish B, Y kg per m² has to be used; so, X kg varnish A have to be compared to Y kg varnish B. Production and use of an article is than evaluated against the “impact categories”, beneath these depletion of resources, emissions into the environment and subsequent impacts like global warming-, acidification-, eutrophication-, and also toxicity-potential. The free program USETox (Rosenbaum et al., 2008[15], 2011[16]) allows the calculation of the health impact of toxic substances emitted to air, water and soil to the general population. The amount emitted is translated into exposure, taking into account the potential fate of the substance between emission from the technical process(es) and uptake by human beings. 

In this paper, the result of a technically feasible substitution process is presented for a wood protecting varnish for outdoor applications against health risk for the worker and the impact category “toxicity” in Life Cycle Assessment. 

The original formulation (Form 1) contained 8 % of naphta 1 and naphtha 2 each (Table 1(Tab. 1)). The alternative solvent composition is shown in Table 2(Tab. 2). For Ethoxypropylacetate and Ethyl-3-ethoxypropionate, classification and labelling data from registration dossiers were cited (European Chemicals Agency, 2016[8]; European Chemicals Agency, 2016[6]). For the other substances, hazard phrases are taken from annex 6, European Union Regulation 1272/2008/EC. The classification according to the global harmonized system (GHS) of the naphtha solvents as mutagens and carcinogens category 1B calls for a substitution already, and Formulation 2 appears to be less hazardous. For naphtha 1 with benzene < 0.1 %, registrants propose a classification as H226 (flammable liquid), H304 (may be fatal if swallowed), H315 (causes skin irritation), H336 (may cause drowsiness and dizziness) and H372 (may affect the central vervous system). For naphtha 2 with benzene < 0.1 %, toluene and n-hexane < 3 %, the registrants propose a classification as H225 (highly flammable liquid) H315, H304 and H336. 

In the following, a closer look on this substitution in terms of toxicological risk assessment on the one hand, and against Life Cycle Impact Assessment on the other hand will be presented. The latter is confined to the impact category “toxicity”, calculated via the publicly available program USETox 2.0 (Rosenbaum et al., 2008[15], 2011[16]). 

The toxicological risk assessment matches NOAEL and NOAECs against exposure levels while applying the varnish, taking a workplace protection point of view. Toxicity in Life Cycle Impact Assessment covers the total emission of substances under investigation, their distribution in the region after release, degradation processes and final intake by the population via inhalation and ingestion of contaminated air, water and food (Rosenbaum et al., 2008[15]).

## Methods

The scenario modelled shall be painting of wooden planks with two types of varnishes which differ in the composition of volatile organic compounds. As the other components of the varnish are unavoidable resins and hardeners which can not be substituted if the required physical properties shall be matched, the whole analysis concentrates on the relative differences made up by the solvents. The new formulation has a higher content of solvents; to apply the same amount of resin and hardener per area, a factor of 1.375 higher mass of the new formulation replaces the old formulation. 10 kg (Formulation 1) or 13.75 kg (Formulation 2) varnish is applied on a surface of 10 m² for 6 h per day; remaining working hours are required for preparation, clean-up etc. The workplace is outdoors in good naturally ventilated areas. The temperature shall be 25° C. 

### Substance data

Substance data concerning environmental behavior and toxicity are taken from the REACH registration dossiers published on the ECHA website. Substance evaluations published by the Deutsche Forschungsgemeinschaft in “The MAK Collection for Occupational Health and Safety” are used as preferred sources for toxicological data. 

### Worker Risk Assessment

The toxicological data of the naphtha solvents as well as for the substitutes will be revisited. With the help of the workers exposure modelling program “Advanced REACH Tool” (ART) (McNally et al., 2014[13]) exposure will be calculated and matched against the derived no effect levels (DNEL) or derived maximum exposure levels (DMEL) and existing official occupational exposure limits in Germany. 

For the ART calculations, the following situation was simulated:

- total exposure time 6 h/d.
- outdoor application, good general ventilation, distant from buildings.
- temperature: 25° C.
- brushing of varnish, less than 1 m away from breathing zone, 1 - 3 m² per hour.
- no carry over from nearby applications.
- Program set to calculate the 75^th^ percentile for the exposure.

### Life Cycle Impact Assessment: Toxicity estimate by USETox

There are several ways to address toxicity in Life Cycle Assessment. The model and program USETox is a consensus model developed by a group of researches under the umbrella of the Society of Environmental Toxicology and Chemistry (SETAC) (Rosenbaum et al., 2008[15], 2011[16]). In this program, the impact category “toxicity” is defined as

CF = XF * FF * EF, ⟺ 

 CF = iF * EF = CTU_h_

where CF is the characterization factor as number of disease cases per kg emitted [cases/kg_emitted_], XF means emission per day [kg/d], FF is the fate factor, covering losses of the emitted substance due to degradation processes [d], EF is the effect factor as number of cases per kg taken up [cases/kg_intake_], iF is the intake fraction [kg_intake_/kg_emitted_], and CTU_h_ are the comparative toxic units, the estimate in increase of morbidity [cases/kg_emitted_].

The fate factor, FF, is estimated with by a multimedia fate model that covers distribution and degradation of the substance in the environment. Several physical-chemical and environmental data are required to simulate the environmental fate of released substances. Molecular mass, vapor pressure, octanol-water partition coefficient, water solubility, degradation in air, water, sediment and soil are required input data to run the USETox model. 

For toxicity, the program USETox considers inhalative and oral uptake and splits non-cancer endpoints from cancer endpoint. Other than in workplace risk assessment, damage is calculated. For doing so, for each substance under consideration an ED_50, h_ has to be defined. The effect factor, EF, is calculated as


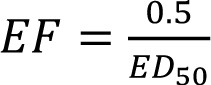


where ED_50,_ is the lifetime dose which causes a 50 % likelihood of disease. The ED_50, h_ is preferably derived from human data, alternatively from data on experimental animals. Details are given in (Rosenbaum et al., 2008[15], 2011[16]). USETox splits the ED_50_ values for cancer and non-cancer disease on the one hand, and oral and inhalation uptake on the other hand. Briefly, for animal data and oral uptake, the procedure is


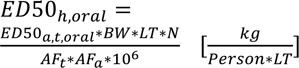


with ED50_a,t,oral_ = Dose (mg/kg/d), for species *a* at *t* exposure time via route *j*; BW: body weight man, 70 kg; LT: life-time, 70 years; N: 365 days per year; AF_t_: extrapolation factor for study duration, AF_t_ = 2 for sub-chronic to chronic and 5 for subacute to chronic extrapolation; AF_a_: Extrapolation animal to man by allometric scaling only. 

For inhalation, the calculation runs as follows:


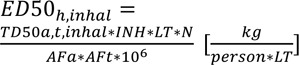


where INH is the daily inhalation volume for man [13 m³/d], and AF_a_ = 1 for inhalation data. For cancer endpoints, ED_50,h_ may be derived from available epidemiologic data by the use of the cancer slope factor. For animal data, the tumorigenic dose TD_50_ shall be taken from the University of Berkeley carcinogen potency data base. As a fall back, the TD_50_ can be derived from available animal data. 

For non-cancer endpoints, ED_50,a _should be taken directly from animal data. If the study data do not provide chronic ED_50_ values, these may be estimated by extrapolation factors, which are 

*ED*_50_ ≈ *NOAEL* * 9,

or, in absence of a NOAEL,

*ED*_50_ ≈ *LOAEL* * 2.5. 

For the route-to-route exposure, extrapolation from inhalation data to oral data can be done by using simply a factor of 1 (Rosenbaum et al., 2011[16]), assuming complete oral absorption. Inhalation data are transformed to oral data by multiplication with the ventilation rate of the species, which is


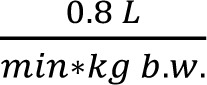


for the rat (European Chemicals Agency, 2012[10]) multiplied with minutes of exposure per day. The result for the complete system under investigation is

*IS* = ∑_i_(CF_i,oral _* *M_i,soil,water_* + *CF_i,inh_* * *M_i,air_*) [cases]

with IS as impact score; estimated increase in morbidity factor in the exposed human population. M_i,j_ is the mass of component i emitted into compartment j. For the estimated number of cases, IS is to be multiplied with the number or individuals per exposed population. 

## Results

### Toxicological Profile of the solvents

#### Naphtha 1 (CAS-No.: 64742-82-1) 

Data for naphtha 1 are available on the European Chemicals Agency (ECHA) web site (European Chemicals Agency, 2016[11]). The MAK Commission of the Deutsche Forschungsgemeinschaft issued a review of naphtha 1 in 2016 (Deutsche Forschungsgemeinschaft, 2016[2]). This group of solvents comprises C9 - C16 aliphatic hydrocarbons, hydrotreated to remove sulfur compounds. In short term exposure tests with volunteers, exposures up to 20 ppm - which was the highest concentration tested - were without effects. The Deutsche Forschungsgemeinschaft (2016[2]) cites a sub-chronic inhalation study where rats were exposed for 6 h/d and 5 d/w against 0, 359, 737 or 1440 pm C10 - C12 paraffin. At the highest dose, animals were lethargic, and a liver weight increase of 40 % was rated as “undesirable”; kidney weight increase in male rats was elevated in all dose groups and is regarded as species specific, but at the top dose also female rats had significantly increased kidney weights. Therefore, the Deutsche Forschungsgemeinschaft (2016[2]) rated 737 ppm as NOAEC. 

In a sub-chronic gavage study rats received 0, 100, 500 or 1000 mg/kg naphtha (free of aromatic compounds, boiling point range 205 - 237° C) on five days per week for 13 weeks (Deutsche Forschungsgemeinschaft, 2016[2]). 500 mg/kg caused increased liver weights, changes in clinical parameters and increased levels of liver enzymes activity in blood. The NOAEL was 100 mg/kg, which is also lower than the oral NOAEL of a two-generation study with rats.

Exposure of gravid rats during gestation days 6 to 15 for 6 h/d revealed a NOAEC of 364 ppm (top dose).

Aromatic free naphtha was negative in the bacterial reverse mutation assay, chromosomal aberration test with CHO cells and the mouse lymphoma assay with and without metabolic activation (Deutsche Forschungsgemeinschaft, 2016[2]). 

After intraperitoneal application, kerosene free of aromatic compounds increased chromosomal aberrations in bone marrow of male, but neither in female B6C3F1 mice nor in SD rats. Dominant lethal test were negative in mice after either s. c. or inhalation application, and negative in rats after i. p. application (Deutsche Forschungsgemeinschaft, 2016[2]). 

Hydrodesulfurated naphtha showed an inconclusive carcinogenic potential in a one year inhalation study with rats and mice with one year post-exposure observation period. Tumor incidences were increased either at the top dose of 5000 mg/m³ only in organs with a high back-ground incidence, or there was no dose-response. 1000 mg/m³ (low dose) caused testical atrophy in male, and islet cell hyperplasia in female mice without a dose-response. 

In a dermal cancer study with mice, hydrodesulfurated naphtha was a promotor, but not an initiator; when applied in higher, irritating concentrations, the solvent caused skin tumors in mice. As the relevance for human beings is not clear, the MAK commission concluded on a classification as carcinogen category 3b (Deutsche Forschungsgemeinschaft, 2016[2]).

Based on the above data, and taking human experience with these and similar solvents into consideration, the DFG issued a MAK-value of 50 ppm = 350 mg/m³ as vapour (Deutsche Forschungsgemeinschaft, 2016[2]).

For a DNEL for consumer inhalation exposure, the NOAEC from a teratogenicity study of 364 ppm is taken as point of departure. A factor of 6 is applied for subacute to chronic exposure, 2.5 for remaining inter-species differences, a factor of 10 for intra-species extrapolation and factors of 24/6 and 7/5 for exposure time corrections. The result is

DNEL_inh, consumer_ = 0.4 ppm = 3 mg/m³.

For the oral DNEL for consumers, the NOAEL of 100 mg/kg of the sub-chronic gavage study is taken as point of departure. The NOAEL is devided by factors of 10 for inter-species and intra-species extrapolation each, and 24/6 and 7/5 to cover daily exposure. A factor of 2 is applied for sub-chronic to chronic extrapolation. The result is

DNEL_oral, consumer_ = 0.09 mg/kg/d.

#### Naphtha 2 (CAS-No.: 64742-95-6)

Data concerning naphtha 2 are available on the ECHA website (European Chemicals Agency, 2016[12]). However, toxicological data looked very similar to those of naphtha 1 (European Chemicals Agency, 2016[11]), which implies that a lot of read across was done; as both types of solvent are different in composition - aromatic free hydrocarbons versus aromatic C8-C10 hydrocarbons - the robustness of this read-across cannot be evaluated by the authors. Therefore, the following summary of toxicological data is based on the evaluations by the DFG for C9 aromatic mixtures (Deutsche Forschungsgemeinschaft, 1998[5], 2001[4]). Rats were exposed against 0, 450, 900 or 1800 mg/m³ C9 aromatic mixture for 6 h/d, 5 d/w for 12 months. The NOEC was 366 mg/m³ (measured concentration). This NOEC is lower than the NOAECs derived from sub-chronic inhalation studies including those addressing neurotoxicity, a three generation inhalation study with rats as well as developmental toxicity studies with rats and mice after inhalation exposure. 

C9 aromatic compounds were negative in bacterial reverse mutation assay, chromosomal aberration, SCE and HPRT assay in CHO cells in vitro. After oral (mice) and inhalation exposure (rats), C9 aromatic compounds did not cause chromosomal aberrations in bone marrow *in vivo*. 

The DFG issued a MAK-value of 20 ppm = 100 mg/m³ (Deutsche Forschungsgemeinschaft, 1998[5]).

For the consumer, the DNEL for inhalation is derived from the chronic inhalation study. Starting with a NOAEC of 366 mg/m³, by applying factors 2.5 for remaining inter-species differences, and 10 * 24/6 * 7/5 (inter-species, consumer, continuous exposure) result in

DNEL_inh, consumer_ = 2.6 mg/m³.

#### Benzene (CAS-No. 71-43-2)

For benzene, ample literature concerning its toxicological profile was published by the United States National Library of Medicine, Integrated Risk Information System (United States Environment Protection Agency, 2016[18]). For cancer, the human unit risk level (one additional case in one million exposed over lifetime) is about 3.5 * 10^-2^ mg/kg/d as oral slope factor, and about 5 * 10^-6^ m³/µg for the inhalation slope factor. From these data, the ED_50, cancer_ values are derived:


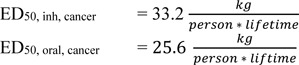


For the non-cancer effects, benzene toxicity on the hematopoietic system as derived from epidemiological studies was taken as the critical endpoint. Inhalation data were transformed to oral uptake data. In a Chinese worker cohort, benzene exposure correlated with reduction in white blood cell, erythrocyte and platelet count (Rothman et al., 1996[17], Table 3(Tab. 3)). The absolute lymphocyte count showed a dose-response, so this effect was chosen to derive the non-cancer ED_50_ values.

Data were modeled with the benchmark dose software version 2.5 (U.S. Environmental Protection Agency). A polynome 2^nd^ degree fitted the data optimal, but it is over-parameterized. However, it represents the worst case as the slope of the curve is steeper in the low dose area than for the linear dose-response curve:

ALC = 1.9 - 0.0248 * D + 0.0002 * D².

The non-cancer ED_50_, now, is defined as the dose that shifts the mean of the ALC to the lower 95 % C. I., the ALC_low_ of the “non-exposed” group. 

ALC_low_ = < *X *> -*t*_95;43 _* σ ⟺

ALC_low_ = 1.9 - 1.68 * 0.4 = 1.23.

This ALC_low_ is linked to an exposure against 40 ppm (129 mg/m³) by the polynomic model. For the ED_50_ it is assumed that exposure takes place for 8 h per day, and the inhalation volume for this time period is assumed to be 10 m³ (worker, low to median activity). The daily uptake, therefore, is 1.29 g/d. As for the endpoint cancer it is assumed that there is no difference in uptake between inhalation and ingestion. As a result, for both exposure paths, the non-cancer ED_50_ for benzene is


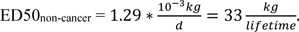


#### n-Butyl acetate (CAS-No.: 123-86-4)

Toxicological data for n-Butyl acetate are published on the ECHA website (European Chemicals Agency, 2016[7]); the toxicological data were also reviewed by the German Deutsche Forschungsgemeinschaft (2003[3]). The latter resource is used for the summary of toxicological data.

Tests with volunteers exposed for 4 h showed clear throat and eye irritation and breathing difficulties at 147 ppm; 15 ppm caused some redness of eyes. 

Rats were exposed against 0, 500, 1500 or 3000 ppm Butyl acetate for 6 h/d for 14 weeks. 500 ppm was the NOEC; at higher concentrations animals showed reduced body weight gain, reduced motor activity and sedation. Organs other than the nervous system were not investigated. 

A two generation study in rats resulted in a NOAEC of 750 ppm for systemic effects. This concentration, however, caused local irritation in the upper respiratory tract (European Chemicals Agency, 2016[6]). 1500 ppm was the NOAEC for teratogenicity in rats, but the LOAEC for maternal and fetal toxicity (Deutsche Forschungsgemeinschaft, 2003[4]). 

Butyl acetate was negative in the bacterial reverse mutation assay and in the *in vitro* chromosomal aberration test (DFG 2003[3]). 

The DFG published a MAK-value of 100 ppm = 480 mg/m³ (Deutsche Forschungsgemeinschaft, 2003[3]).

For the consumer, the DNEL for inhalation is derived from the sub-chronic inhalation study. Starting with a NOEC of 500 ppm, by applying factors of 2 for subacute to chronic exposure, 2.5 for remaining inter-species differences, 10 * 24/6 * 7/5 (inter-species, consumer, continuous exposure) result in

DNEL_inh, consumer_ = 1.8 ppm = 8.6 mg/m³.

#### Ethoxypropylacetate (CAS-No.: 54839-24-6)

Rats were exposed against 0, 100, 300 or 1200 ppm Ethoxypropylacetate for 6 h/d, 5 d/w for 4 weeks (Deutsche Forschungsgemeinschaft, 2007[1]). The middle and high concentration caused altered reactions on external stimuli; the NOEC was 100 ppm (600 mg/m³). Probably the same study is cited on the ECHA website (European Chemicals Agency, 2016[8]). Delayed reactions to external stimuli were transient in the middle dose group and persisted during exposure only in the top dose. The top dose of 1176 ppm (measured concentration) was said to be the NOAEC due to lack of any histopathological, clinical chemistry and hematological effects. 

1-Ethoxy-2-propylacetate was negative in the bacterial reverse mutation assay and in the *in vitro* chromosomal aberration test with/out metabolic activation (Deutsche Forschungsgemeinschaft, 2007[1]).

The substance is expected to hydrolyze rapidly *in vivo*; the resulting 1-Ethoxy-2-propanol is not toxic to development (Deutsche Forschungsgemeinschaft, 2007[1]). 

The DFG published a MAK-value of 50 ppm = 300 mg/m³ (Deutsche Forschungsgemeinschaft, 2007[1]).

For the general population, the DNEL for inhalation is derived from the subacute inhalation study. Starting with a NOAEC of 1200 ppm, by applying factors of 6 for subacute to chronic exposure, 2.5 for remaining inter-species differences, 10 * 24/6 * 7/5 (inter-species, consumer, continuous exposure):

DNEL_inh, consumer_ = 8.4 mg/m³.

#### Ethyl-3-ethoxypropionate (CAS-No.:763-69-9)

Toxicological data for Ethyl-3-ethoxypropionate can be retrieved from the ECHA website (European Chemicals Agency, 2016[6]). In an oral gavage study, rats received 0, 100 or 1000 mg/kg Ethylethoxy-propionate 5 d/w for 4 weeks. 100 mg/kg was the NOEL. 1000 mg/kg caused slight to moderate increase in aspartate amino transferase, alanine amino transferase, creatinine and sorbitol dehydrogenase in blood. In the absence of histopathological correlates, the authors rate this dose level as NOAEL. 

In a sub-chronic inhalation study, rats were exposed against 0, 250, 500 or 1000 ppm Ethylethoxypropionate 6 h/d, 5 d/w. For male rats, body weight was reduced by 5 %, 10 % and 15 % for the low, middle and high dose group, respectively. Due to absence of histopathological lesions, the authors rate 500 ppm (3000 mg/m³) as NOAEC. 

In a teratogenicity study, pregnant rats were exposed against 125, 250, 500, and 1000 ppm Ethylethoxypropionate during gestation days 6 to 15. The NOAECs for maternal toxicity, fetotoxicity and teratogenicity were 250, 500 and 1000 ppm respectively. 

Ethylethoxypropionate was not mutagenic in the bacterial reverse mutation assay and the chromosomal aberration assay and HPRT assay in CHO cells.

For the inhalation route, the registrants of Ethylethoxypropionate propose a DNEL of 610 mg/m³ for workers and 72.6 mg/m³ for the general population, based on irritation effects. However, 500 ppm caused a 10 % body weight decrease in the sub-chronic inhalation study in males, and in the teratogenicity study, 250 ppm were identified as maternal NOAEC; for that reason, 250 ppm - the NOEC of the sub-chronic study - should be taken as starting point for the DNEL. For workers, a factor of 2.5 is used for remaining inter-species differences (toxicodynamic factor rat to man), a factor of 5 (worker) or 10 * 24/6 * 7/5 (consumer) for intra-species extrapolation and a factor of 2 for sub-chronic to chronic extrapolation. The results are 

DNEL_inh, worker _ = 120 mg/m³;

DNEL_inh, consumer_ = 11 mg/m³.

For oral uptake, the DNEL for the general population is 1.2 mg/kg/d (assessment factor 600 for the subacute oral NOAEL, multiplied by 5/7 for daily exposure).

### Worker Risk Assessment

Exposure estimates for the different solvents as delivered by the Advanced REACH Tool (McNally et al., 2014[13]) are summarized in Table 4(Tab. 4). For the naphta solvents 1 and 2, due to the classification and labeling provided in the safety data sheets, the content of benzene must be below 0.1 %. From the production of the solvents it is to be expected that some benzene is present. As the actual content of benzene in naphtha 1 and 2 is unknown to the authors, the benzene content is estimated as 0.05 % in the respective naphtha solvent, which makes 0.008 % in the varnish (0.16 * 0.05 %).

Based on calculated median exposure concentrations, for the two different formulations, the Hazard Index


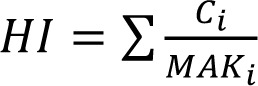


is 0.046 for solvent Formulation 1 and 0.037 for solvent Formulation 2. However, if benzene is present at about 0.008 % in Formulation 1, the calculated mean exposure against 0.64 µg/m³ benzene would give reason to an additional cancer incidence of 32 cases in 10000 persons exposed over lifetime. 

### Life Cycle Assessment, impact category toxicity

The intake fraction (iF), fate and distribution of a substance is calculated with the program USETox. Measured data on ready biodegradability were available for all compounds, and these were transformed to aquatic halves lives according to the REACH Guidance (European Chemicals Agency, 2014[9]). The naphtha components were readily degradable, but missed the 10 d windows. Degradation rates in air, sediment and soil were calculated with the US EPA Episuite program version 4.1 (United States Environment Protection Agency, 2016[19]). For the naphta products, a representative molecule had to be chosen for the EPISUITE program to run, which was decane for naptha 1, and 4-ethyl-methylbenzene for naphta 2. N-Butylacetate was listed in the USETox 2.0 database already. For the remaining substances, data are summarized in Table 5(Tab. 5). Due to the toxicological profiles described in chapter 3.2, ED_50 _values were derived for non-cancer endpoints, only (Table 6(Tab. 6)) - with the exemption of benzene. As either 10 kg Formulation 1 or 13.75 kg Formulation 2 are applied per day, the solvents contained are assumed to be emitted within one day. The characterization factor CF, cases per kg emitted, were calculated for emission into urban air in Europe. The results are listed in Table 7(Tab. 7). 

The total proportion of disease cases in a population can be estimated by summing up the emissions over all working days in 70 years life time - 240 working days per year - multiplied by the substance specific characterization factors. 


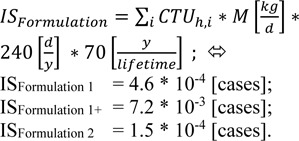


It is debatable whether or not benzene has to be included into Formulation 1. As a matter of fact, benzene might be present in naphtha 1 and naphtha 2 due to their origin. As the products were not labeled as carcinogenic and mutagenic, the content must be below 0.1 %. If benzene is present at 0.008 % (Formulation 1+), the impact score for toxicity of the designed operation increases by a factor of about 16.

## Discussion

The comparative toxicity of two formulations of a reactive varnish that differ in the qualitative and quantitative content of solvents was assessed. This assessment was done against workplace risk assessment on the one hand, and the Life Cycle Assessment Impact Category “Toxicity” on the other hand. The exposure scenario was defined as spreading 10 kg (Formulation 1) or 13.75 kg (Formulation 2) varnish over 10 m² during 6 h per working day.

For the worker risk assessment, when the potential content of benzene in Formulation 1 is not covered, in general both formulations can be used in a safe way for the exposure scenario outlined. Individual exposure limits are not exceeded, and also the combined exposure is not expected to cause considerable damage to health. “What if” scenarios might be discussed, for example concerning adverse outcomes in accidental situations where the exposure limits are not met. Both formulations contain substances that are labeled as causing drowsiness and dizziness and skin irritation. The naphtha formulation components are additionally classified as fatal if swallowed - which should not be a realistic problem at workplaces - and as affecting the nervous system. The latter might be regarded as relatively critical, which calls for a substitution of Formulation 1 by Formulation 2. 

If benzene is present at 0.008 % in Formulation 1, the calculated exposure is 0.64 (median or 50-percentile) or 1.3 mg/m³ (75-percentile). Robbins et al. (2013[14]) demonstrated that wiping exercises with solvents containing 0.1 % benzene led to a measured exposure of about 0.49 mg/m³, and the ratio of calculated to measured exposure ranged from 0.42 to 2.1. Exposure against 1.3 mg/m³ benzene is related to an excess cancer incidence of 32 in 10000 exposed persons. For carcinogens, the DMEL according to the ECHA guidance document (European Chemicals Agency, 2012[10]) shall not exceed 1 case in 100,000 to 1 million exposed. Therefore, this aspect would call for a substitution of Formulation 1 against Formulation 2. This result would also call for specific limits for benzene in formulations for classification and labelling below the current European Union generic limit of 0.1 %.

In Life Cycle Assessment, toxicity is one of the potential impact categories that can be addressed. One way to do so is provided by the publicly available program USETox (Rosenbaum et al., 2008[15], 2011[16]). There are certain differences to the workplace risk assessment: the compound under investigation is emitted into environmental compartments; in these environmental compartments it undergoes distribution and degradation processes; depending on distribution and degradation processes, certain concentrations will show up in air, drinking water and food, and finally be taken up by inhalation and ingestion; the model than calculates potential damage as disease cases per kg substance emitted. The fate of the emitted substance is estimated with multimedia environmental models. The choice of a region addresses the different environmental parameters that dictate the degradation of a substance, like sunlight intensity, rain and temperature; the program makes use of the mean values of climate parameters in the region. Toxicity is translated into disease cases per kg emitted. Disease is split up in “cancer” and “non-cancer” without further detail. As there are uncertainties concerning the environmental fate as well as concerning extrapolation factors in toxicity and subsequent disease, the final outcome is appropriately called “potential” cases per kg; it is more are ranking tool for substances and not meant to be a prediction in expected disease incidence. For the substance ranking, a difference of up to a factor of 1000 is not necessarily an indicator for detectable higher / lower toxicity (Rosenbaum et al., 2008[15]). Against that background, the differences between Formulation 1 (with and without benzene) and Formulation 2 are not convincing for a substitution. Further, for a complete picture of the toxicity potential in Life Cycle Assessment, the upstream processes, e. g. toxicity potentials of emissions due to the production processes of the solvents, need to be covered as well. This would require the (public) availability of Life Cycle Inventory data of all upstream processes, which is the case for the naphtha solvents only. 

Over all, for workplace safety reasons the substitution of Formulation 1 by Formulation 2, as the presence of benzene is likely, is recommended. Such a substitution would also lower the toxicity potential in the Life Cycle Assessment of the product. 

## Conflict of interest

Guenter Kirstein is CEO of ALGURA Chemie GmbH. The formulation investigated is a product of this company. Philipp A. Georg got a financial support for lab work from ALGURA Chemie GmbH. Thomas Schupp and Philipp A. Georg declare no conflict of interest. 

## Figures and Tables

**Table 1:**
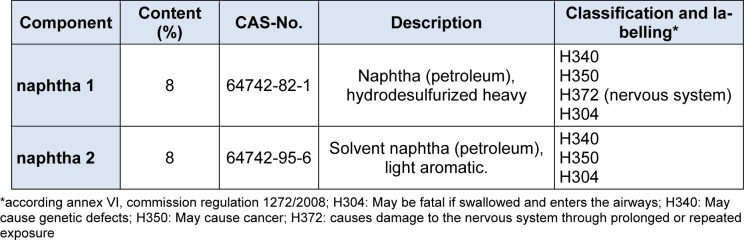
Formulation 1 (Form 1), the original solvent formulation

**Table 2:**
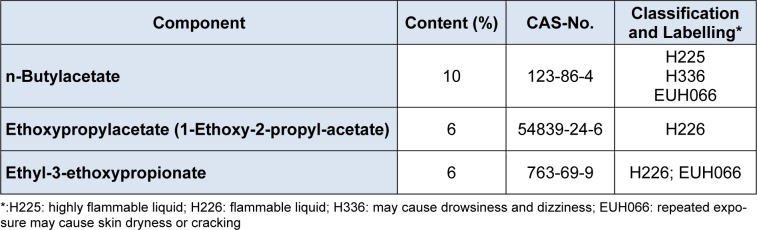
Formulation 2 (Form 2), the alternative solvent formulation

**Table 3:**
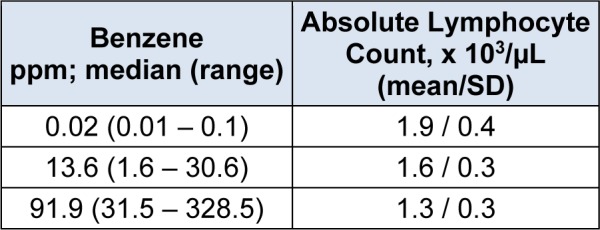
Dose-response of absolute lymphocyte count against benzene exposure in a Chinese cohort (Rothman et al*.,* 1996)

**Table 4:**
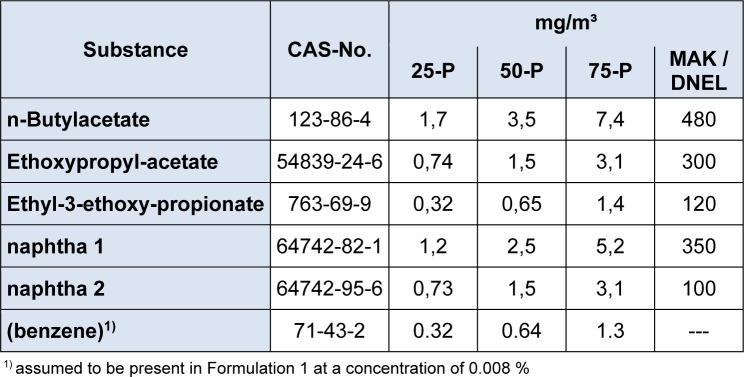
25-, 50- and 75-percentiles of exposure estimates for workers handling the reactive varnish, calculated with the Advanced REACH Tool (ART)

**Table 5:**
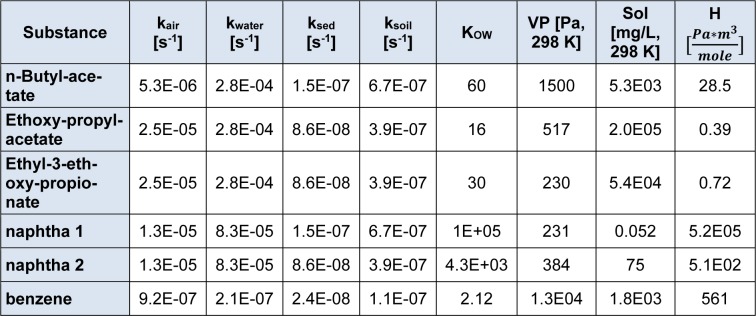
First order degradation rate constants in air, water, sediment and soil, octanol-water partition coefficient, vapor pressure, solubility in water and Henry constant

**Table 6:**
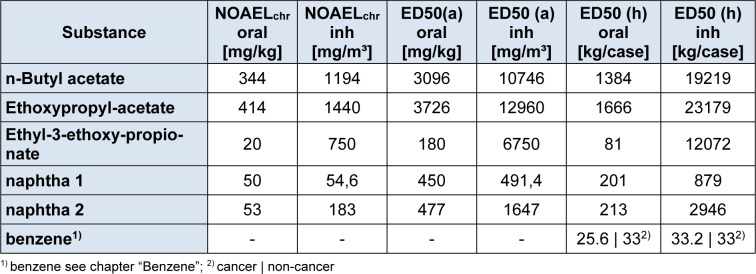
ED_50_ values of the solvents

**Table 7:**
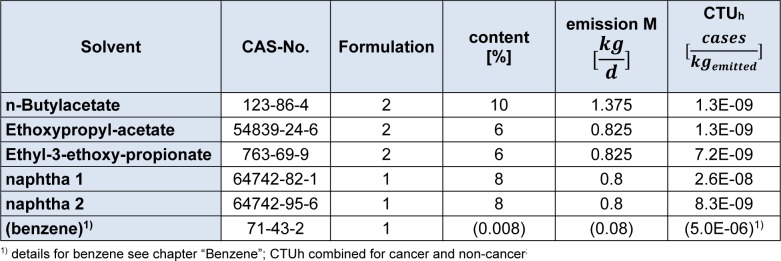
Emission into the air per day, depending on formulation
